# A case of antidromic atrioventricular reciprocating tachycardia via the atriofascicular pathway with suspected minor manifest fusion during ventricular pacing

**DOI:** 10.1002/joa3.12747

**Published:** 2022-06-24

**Authors:** Hiroyuki Kamada, Satoshi Nagase, Koji Miyamoto, Takeshi Aiba, Kengo Kusano

**Affiliations:** ^1^ Department of Cardiovascular Medicine and Hypertension Kagoshima University Graduate School of Medical and Dental Sciences Kagoshima Japan; ^2^ Department of Advanced Arrhythmia and Translational Medical Science National Cerebral and Cardiovascular Center Suita Japan; ^3^ Department of Cardiovascular Medicine National Cerebral and Cardiovascular Center Suita Japan

**Keywords:** atriofascicular pathway, atrioventricular reciprocating tachycardia, entrainment, fusion, Mahaim fiber

## Abstract

In this case with antidromic atrioventricular reciprocating tachycardia via the atriofascicular pathway, entrainment from the right ventricular apex showed minor constant fusion. This may indicate that an atriofascicular pathway with distal arborization can connect to the branch of the right bundle and partly to the working myocardium.

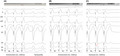

A 16‐year‐old man who had recurrent episodes of palpitation was referred to our center. A 12‐lead electrocardiogram recorded during sinus rhythm showed no delta waves and one recorded during palpitation showed left bundle branch block‐type wide QRS tachycardia with a heart rate of 169/min (Figure 1A,B). During an electrophysiological study, multielectrode catheters were placed in the free wall of the right atrium (RA), His bundle region, coronary sinus, and right ventricular apex (RVA). During sinus rhythm, the cycle length (CL), atrio‐His interval, and His‐ventricular interval (HV) were 760, 81, and 39 ms, respectively. Atrial extrastimulus caused prolongation of the A‐QRS interval, shortening of the HV interval, and prolongation of the QRS interval. The His bundle potential, which had initially excited in the anterograde direction, changed to be in the retrograde direction. The earliest site of retrograde atrial excitation was only in the His region, and the response of para‐Hisian pacing showed a pattern of atrioventricular node conduction. The left bundle branch‐type tachycardia with a CL of 354 ms was easily inducible with atrial and ventricular pacing. The order of atrial excitation during tachycardia was like that of retrograde conduction during ventricular stimulation, with the His bundle region being the earliest site of excitation. The excitation of the His bundle potential was in the retrograde direction. Moreover, the right bundle branch potential confirmed during sinus rhythm preceded the local ventricular potential, QRS wave, and His bundle potential during tachycardia (Figure 1C,D). During tachycardia with a CL of 356 ms, entrainment was performed from the lateral wall of the RA with a CL of 340 ms. The QRS complex formed with a long conduction time was consistent with that during tachycardia, and no fusion was observed (Appendices). A single atrial stimulus from the lateral wall of the RA at the timing of the His refractory period, which did not antidromically capture the atrial muscle in the atrioventricular junction region, reset the tachycardia advancing the timing of the ventricular and subsequently atrial activation orthodromically with a long conduction time (Figure 2). During tachycardia with a CL of 354 ms, entrainment pacing from the RVA with a CL of 340 ms showed a post‐pacing interval (PPI) of 362 ms, and the PPI minus tachycardia CL was 8 ms. These findings were considered strongly suggestive of antidromic atrioventricular reciprocating tachycardia (AVRT) over an atriofascicular pathway rather than atrioventricular nodal reentrant tachycardia with a bystander accessory pathway. Despite AVRT over an atriofascicular pathway, entrainment from the RVA showed minor but manifest fusion (Figure 3). Radiofrequency application at the anterolateral region of the tricuspid annulus successfully eliminated the atriofascicular pathway. After radiofrequency ablation, the tachycardia was no longer inducible.

**FIGURE 1:**
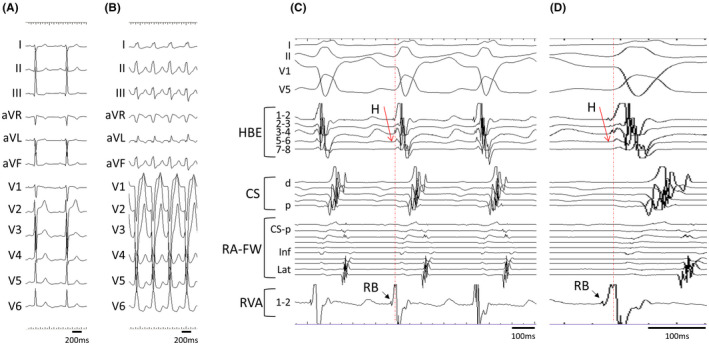
(A) ECG during sinus rhythm. (B) ECG during tachycardia. (C, D) intracardiac electrograms during tachycardia. (D) Is an enlargement of a part of (C). During tachycardia, the right bundle potential (RB) preceded the local ventricular potential, QRS complex, and His bundle potential (H). Therefore, the ventricular end of the accessory conduction pathway should connect to the right bundle branch. Red arrows indicate the retrograde direction of excitation of the His bundle potential. CS, coronary sinus; d, distal; HBE, his bundle electrogram; inf, inferior; Lat, lateral; p, proximal; RA‐FW, free wall of the right atrium; RVA, right ventricular apex.

**FIGURE 2:**
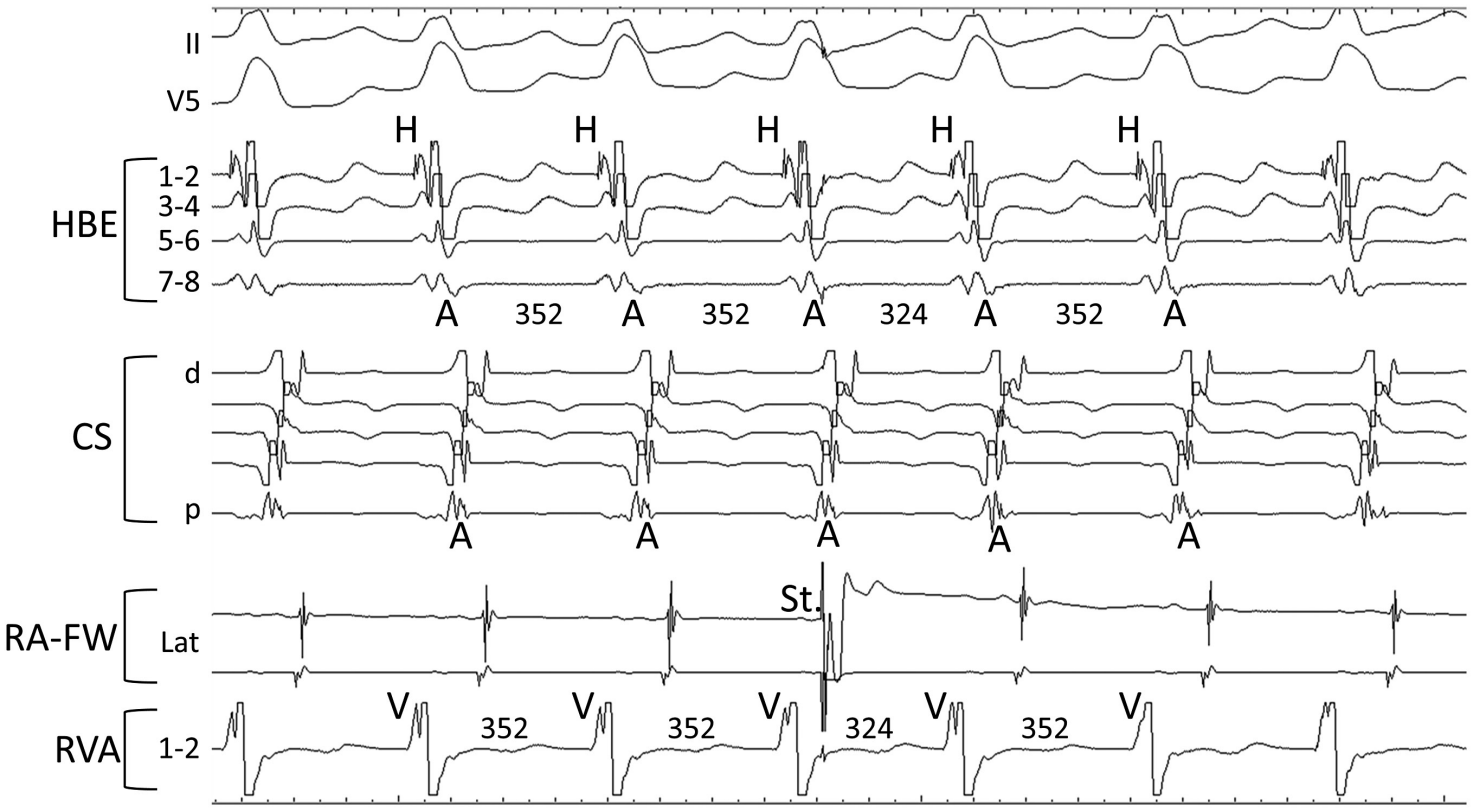
A single atrial stimulus from the lateral RA‐FW during the his refractory period captured the ventricle and subsequently reset the tachycardia.

**FIGURE 3:**
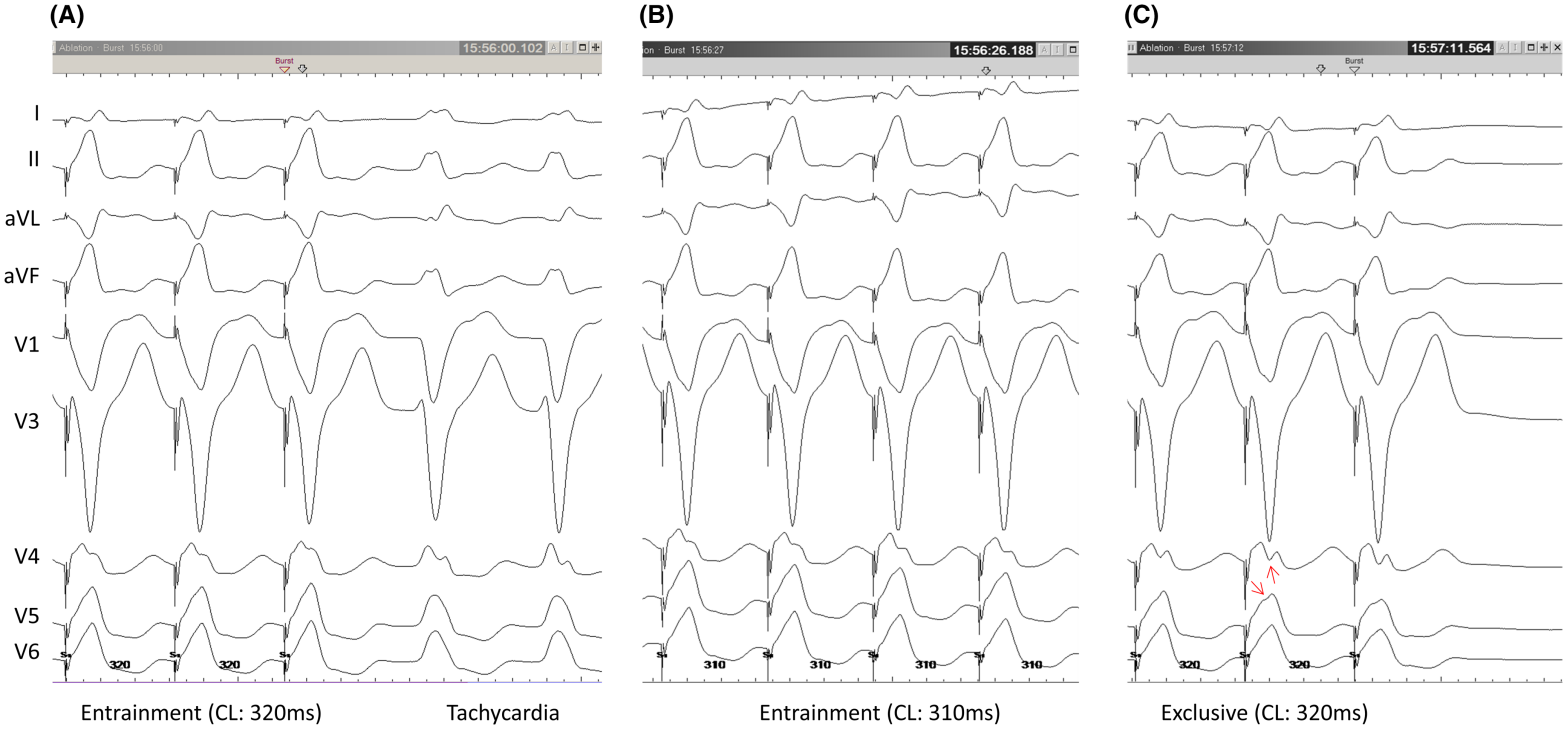
Entrainment from the RVA showed minor constant fusion. (A) Entrainment pacing with a cycle length of 320 ms followed by tachycardia. (B) Entrainment pacing with a cycle length of 310 ms. (C) Exclusive pacing with a cycle length of 320 ms during sinus rhythm immediately after entrainment study at the same RVA site. Considering the effects of hemodynamics and respiratory status, the pacing study was performed consecutively and speedily as shown by the time description. Red arrows during exclusive pacing indicate different QRS morphologies from those during entrainment pacing.

Mahaim fiber was first described by Mahaim as a tissue leading from the atrioventricular node to the ventricles in 1938.^1^ Nowadays, the term Mahaim fibers is generally used to describe an antegrade‐only conducting accessory pathway with decremental conduction properties that are typically right‐sided.^2^ In this case, as mentioned above, the findings of electrophysiological studies led to a diagnosis of antidromic AVRT via an atriofascicular Mahaim pathway. During tachycardia, the right bundle potential was recorded before the local ventricular potential, QRS complex, and His bundle potential. Therefore, the ventricular end of the accessory conduction pathway was diagnosed as the right bundle branch. In general, antidromic AVRT via an atriofascicular pathway is considered to have no fusion because of collision within the specialized conduction system. However, minor manifest fusion during the overdrive pacing from the RVA was confirmed in this case although no fusion was observed from the RA. It is thought that the atriofascicular pathway inserts into the moderator band at or adjacent to the right bundle branch.^3^ Ho S.Y. reported that the atriofascicular pathway connects with the “distal ramifications” of the right bundle branch.^4^ But, the distal insertion of the atriofascicular pathway is less well understood. Certainly, fusion will not occur when the atriofascicular pathway is directly and solely connected to the right bundle branch without distal arborization. But manifest fusion could occur when the atriofascicular pathway with distal arborization connects to the branch of the right bundle and partly to the working myocardium (Figure 4). In fact, it has been reported that fused atriofascicular potentials and distal right bundle branch potentials are widely seen on activation maps in cases of antidromic AVRT via the atriofascicular tract.^5^ Actually, we could not deny the possibility that rapid ventricular pacing accompanied by significant movement of the heart and thorax affected the QRS morphology. Moreover, a possible fusion area in the working myocardium should be limited. However, the electrophysiological observations in this case, including the findings of previous papers, suggest that the atriofascicular pathway does not connect solely to the right bundle but a branch of the right branch with distal arborization.

**FIGURE 4:**
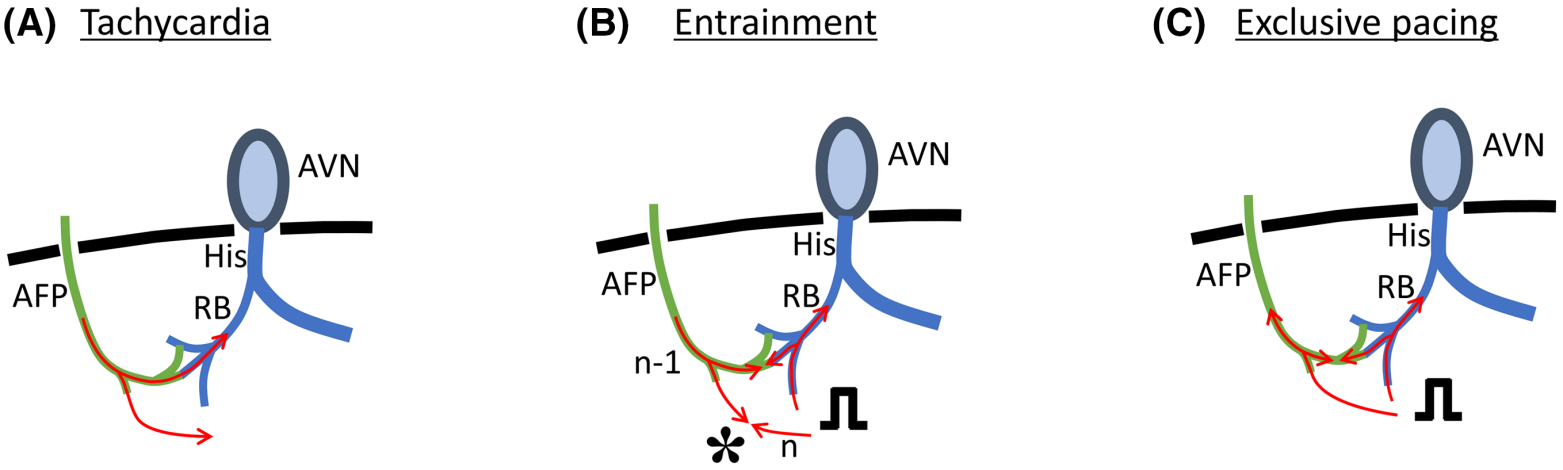
Schematic representation of the reentrant circuit of the tachycardia (A), expected collision site (*) during entrainment pacing from the RVA (B), and exclusive pacing from the RVA (C). Atriofascicular pathway (AFP) with distal arborization can connect to the branch of the right bundle (RB) and partly to the working myocardium, which will cause minor manifest fusion during entrainment. AVN, atrioventricular node.

## CONFLICT OF INTEREST

Dr. Satoshi Nagase is affiliated with a department endowed by Japan Medtronic Inc. The remaining authors have nothing to disclose.

## Supporting information


Appendices
