# Supplementary figures and images for: A case of antidromic atrioventricular reciprocating tachycardia via the atriofascicular pathway with suspected minor manifest fusion during ventricular pacing
Source: J Arrhythm. 2022 Jun 24;38(4):656–9. doi: 10.1002/joa3.12747 (PMC9347201; doi:10.1002/joa3.12747)

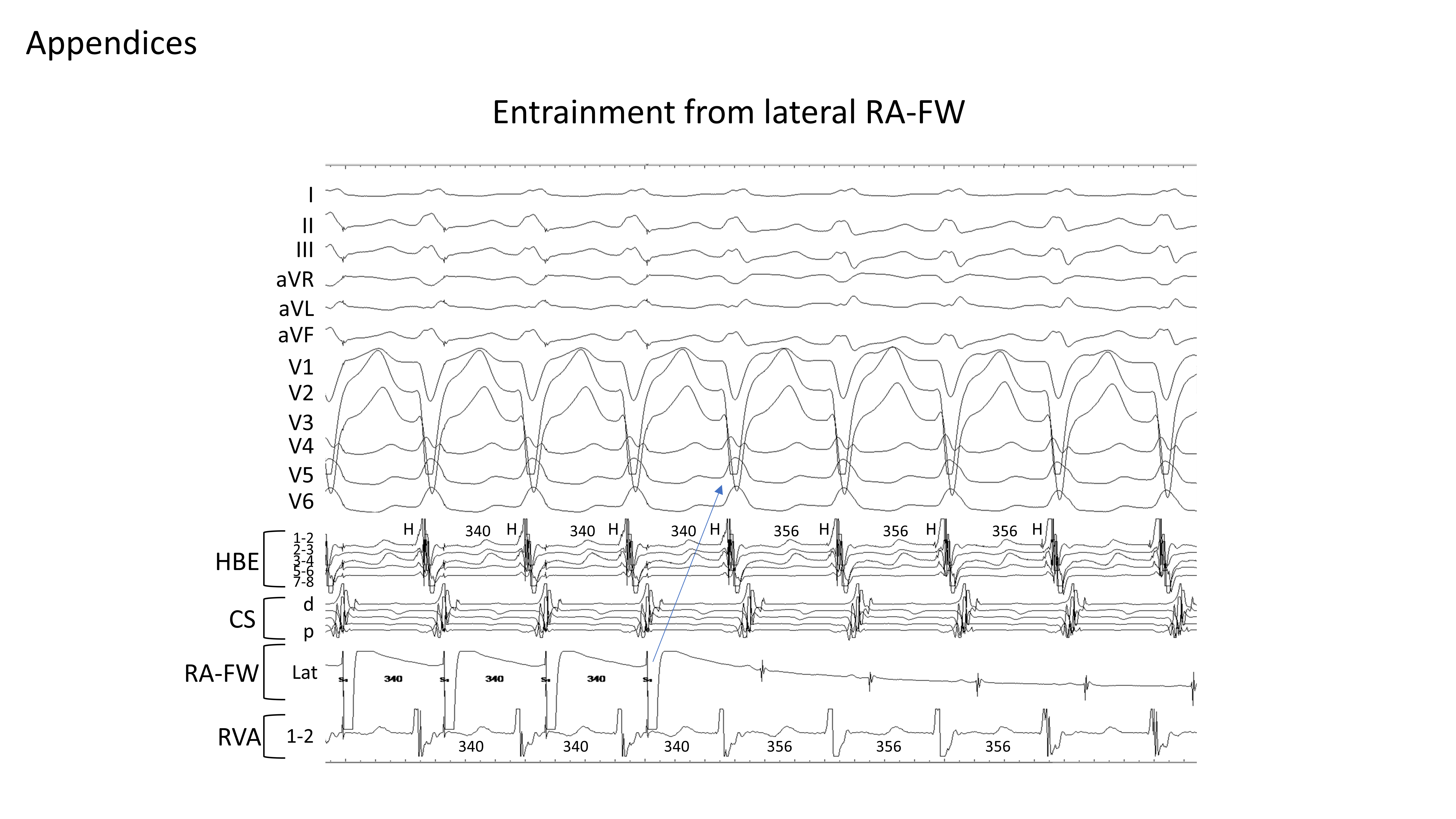

Supplement: Supplementary file 1 — Appendices [file JOA3-38-656-s001.TIF]
